# Complex effects of high-flow nasal cannula therapy on hemodynamics in the pediatric patient after cardiac surgery

**DOI:** 10.1186/s40560-017-0227-y

**Published:** 2017-05-30

**Authors:** Yu Inata, Muneyuki Takeuchi

**Affiliations:** Department of Intensive Care Medicine, Osaka Women’s and Children’s Hospital, 840 Murodo-cho, Izumi, Osaka 594-1101 Japan

**Keywords:** High-flow nasal cannula, Congenital heart disease, Cardiopulmonary interactions

## Abstract

**Background:**

The high-flow nasal cannula (HFNC) system has been widely used for children in various clinical settings. However, the physiological and clinical impact of HFNC therapy on the pediatric patient with respiratory distress after cardiac surgery has not been thoroughly investigated.

**Main body of the abstract:**

It seems logical to use HFNC as a primary therapy for post-extubation respiratory failure after congenital heart surgery, in which low cardiac output syndrome and upper airway obstruction are commonly encountered; the HFNC therapy alleviates the work of breathing and large negative swings of intrathoracic pressure, which in turn helps to decrease the systemic ventricular afterload. When applying HFNC to patients after congenital heart surgery, however, consideration must be given to its diverse effects on hemodynamics because of the complex respiratory and cardiac pathophysiology in these patients. The positive pressure generated by HFNC can exert different effects on pulmonary vascular resistance depending on the lung condition, while its impact on cardiac output may also differ depending on the cardiac physiology. The hemodynamic effects of HFNC may become even more complex in a patient with a single ventricle. To better assess its physiologic effects in patients after cardiac surgery, future studies could utilize various modalities including esophageal balloon catheters, electrical impedance tomography, and near-infrared spectroscopy. At the same time, studies should focus on specific types of cardiac pathophysiology or surgery when evaluating the effects of HFNC, since it may exert various effects, depending on the cardiac physiology or preoperative pulmonary hemodynamics. Lastly, the optimal flow rate at which the benefit of HFNC is maximized through favorable cardiopulmonary interactions should be determined in future studies.

**Short conclusion:**

Further studies are needed to better understand the effect of HFNC in different cardiac and respiratory physiologies, given their complexity in pediatric patients after cardiac surgery.

## Background

The high-flow nasal cannula (HFNC) system has gained great popularity owing to several advantages: greater comfort and better tolerability [1], improved mucociliary clearance [2], decreased nasal airway resistance [3], a “wash-out” effect of dead space [4], generation of positive airway pressure [5], and ability to reliably deliver fixed concentrations of oxygen and other therapeutic gases, including inhaled nitric oxide [6]. When compared to noninvasive positive-pressure ventilation with a tight-fitting mask, the greater comfort and tolerability of HFNC becomes a great advantage in otherwise uncooperative children. Indeed, the HFNC therapy has been widely used for children in various clinical scenarios [7–9]. Despite the widespread use of HFNC in the pediatric intensive care unit, the physiological and clinical impact of HFNC therapy on the pediatric patient with respiratory distress after cardiac surgery has not been thoroughly investigated [10].

## Main text

It seems logical to use HFNC as a primary therapy for post-extubation respiratory distress after pediatric cardiac surgery. First, low cardiac output syndrome (LCOS) is commonly encountered after congenital heart surgery and contributes to significant postoperative morbidity and mortality [11]. In the presence of LCOS, increased work of breathing (WOB) is poorly tolerated; excessively negative intrathoracic pressure increases systemic ventricular afterload, while increased diaphragmatic oxygen consumption reduces oxygen delivery to vital organs [12, 13]. In this situation, HFNC therapy helps to decrease afterload by providing positive pressure and by abating sympathetic nervous system activity via unloading of respiratory and cardiovascular work [5, 12]. Second, an upper airway obstruction (UAO) is relatively common after pediatric cardiac surgery and has been implicated as an important contributor to extubation failure in this population [14–16]. UAO increases negative swings of intrathoracic pressure and increases WOB, thereby imposing an undue burden on a recovering myocardium and further complicating LCOS; HFNC therapy could alleviate the WOB and large negative swings of intrathoracic pressure.

In addition to these advantages, however, consideration must be given to its diverse effects on hemodynamics in patients after congenital heart surgery, given the complex respiratory and cardiac pathophysiology in these patients. The positive pressure generated by HFNC can exert different effects on pulmonary vascular resistance (PVR), depending on the lung condition; it may increase PVR as lung volumes rise above functional residual capacity (FRC) in a patient with healthy lungs, but may decrease PVR by restoring FRC and improving alveolar oxygen tension in a patient with pulmonary edema or atelectasis [12]. Furthermore, the effects of positive pressure on cardiac output (CO) may also differ depending on cardiac function. When the systolic function of the systemic ventricle is impaired, positive pressure by HFNC likely improves CO, as the beneficial effect on systemic ventricular afterload outweighs the potential negative impact on venous return. In a patient with preserved systolic function, however, the negative effects of decreased venous return may predominate, leading to reduced CO [12].

The effects of HFNC may become even more complex in a patient with a single ventricle [17]. In parallel circulation, for example, reduction of PVR through maintenance of FRC by HFNC could inadvertently increase the ratio of pulmonary flow to systemic flow, while the benefit of reducing oxygen demand by respiratory muscles and reducing systemic vascular resistance through alleviation of sympathetic nervous system stimulation may outweigh the potential disadvantages. Following the Fontan procedure, the negative effects of positive pressure on venous return and ventricular filling typically predominate over the positive effects on the afterload of the systemic ventricle [12]. However, when the systolic function is severely depressed, positive pressure may be beneficial, by decreasing afterload and oxygen consumption. In addition, it may decrease PVR by restoring and maintaining FRC in the presence of pulmonary edema or atelectasis.

In this issue of the *Journal*, Shioji et al. reported the physiological impact of HFNC therapy on post-extubation acute respiratory failure in children after various cardiac surgeries. They compared respiratory and hemodynamic parameters before and 1 h after applying HFNC. HFNC therapy significantly decreased respiratory rate, as expected from a physiological study in healthy volunteers [5] and other studies in adults and children with respiratory distress [18, 19]. Interestingly, systolic blood pressure (SBP) in the patients with serial circulation significantly decreased after HFNC therapy. We speculate that decreased SBP is a result of reduction in sympathetic activity via unloading respiratory and cardiovascular work, given the fact that heart rate also decreased, albeit not significantly. While the authors used only rudimentary respiratory and hemodynamic parameters when assessing the effect of HFNC, they should be highly commended for their efforts to describe the physiological impact since the single previous report on HFNC therapy in this population examined only changes in arterial blood gas parameters [10]. Nonetheless, to better design a future study and deepen our understanding, we can gain some insight from previous studies that have utilized various techniques to assess respiratory and hemodynamic parameters. For example, an esophageal balloon catheter and electrical activity of the diaphragm have been utilized to investigate the effects of HFNC on WOB [20, 21]. Parke et al. used electrical impedance tomography to assess end-expiratory lung volume with varying degrees of gas flow [5]. Regarding cardiovascular parameters, near-infrared spectroscopy was used to evaluate cerebral circulation while applying HFNC in a patient with Fontan circulation [22]. Although these modalities to assess respiratory and cardiovascular physiology have their own limitations, they certainly augment the assessment of physiological impact.

The authors also attempted a subgroup analysis of the effect of HFNC based on the cardiac physiologies (serial circulation vs. single ventricle). Besides the limitation of the small sample size, future studies could be improved from other aspects as well. First, the patients could be classified into specific subgroups, not simply into serial circulation and single ventricle groups, by clearly delineating the types of surgery and original cardiac physiology. HFNC therapy may exert different effects depending on the cardiac physiology—systemic to pulmonary shunt vs. bidirectional Glenn operation vs. Fontan operation—or, depending on the preoperative pulmonary hemodynamics, high vs. low pulmonary flow. Habre et al. demonstrated that immediate improvement in lung function after surgical repair of congenital heart disease was seen in children with high pulmonary flow, but not with restricted pulmonary flow [23]. It would be interesting to know if HFNC therapy exerts different respiratory and hemodynamic effects in these contrasting types of pulmonary circulation. Lastly, the flow rate should be specified, e.g., 2 L/kg/min, or changed according to an a priori protocol, not at the discretion of bedside physicians. Given the evidence of progressive increase in nasopharyngeal airway pressure and end-expiratory lung volume with a higher flow rate [5], there is likely a flow rate at which the benefit is maximized through optimal cardiopulmonary interactions. Such an optimal flow rate should be sought in future studies.

## Conclusion

Further studies are needed to better understand the effect of HFNC in different cardiac and respiratory physiologies, given their complexity in pediatric patients after cardiac surgery. In the meantime, we should be cognizant of both potential positive and negative effects on hemodynamics when applying and titrating HFNC in this population.

## Abbreviations

HFNC: High-flow nasal cannula
LCOS: Low cardiac output syndrome
WOB: Work of breathing
PVR: Pulmonary vascular resistance
CO: Cardiac output
SBP: Systolic blood pressure

## References

[1] Roca O, Riera J, Torres F, Masclans JR (2010). High-flow oxygen therapy in acute respiratory failure. Respir Care.

[2] Hasani A, Chapman TH, McCool D, Smith RE, Dilworth JP, Agnew JE (2008). Domiciliary humidification improves lung mucociliary clearance in patients with bronchiectasis. Chron Respir Dis.

[3] Fontanari P, Burnet H, Zattara-Hartmann MC, Jammes Y (1996). Changes in airway resistance induced by nasal inhalation of cold dry, dry, or moist air in normal individuals. J Appl Physiol.

[4] Möller W, Feng S, Domanski U, Franke KJ, Celik G, Bartenstein P (2017). Nasal high flow reduces dead space. J Appl Physiol.

[5] Parke RL, Bloch A, McGuinness SP (2015). Effect of very-high-flow nasal therapy on airway pressure and end-expiratory lung impedance in healthy volunteers. Respir Care.

[6] Fearnley RA, Gatward JJ, Gattas DJ, Wales NS (2014). Combination of high-flow nasal cannula oxygen therapy and inhaled nitric oxide in a paediatric patient with respiratory distress. Anaesth Intensive Care.

[7] Wing R, James C, Maranda LS, Armsby CC (2012). Use of high-flow nasal cannula support in the emergency department reduces the need for intubation in pediatric acute respiratory insufficiency. Pediatr Emerg Care.

[8] McKiernan C, Chua LC, Visintainer PF, Allen H (2010). High flow nasal cannulae therapy in infants with bronchiolitis. J Pediatr.

[9] Byerly FL, Haithcock JA, Buchanan IB, Short KA, Cairns BA (2006). Use of high flow nasal cannula on a pediatric burn patient with inhalation injury and post-extubation stridor. Burns.

[10] Testa G, Iodice F, Ricci Z, Vitale V, De Razza F, Haiberger R (2014). Comparative evaluation of high-flow nasal cannula and conventional oxygen therapy in paediatric cardiac surgical patients: a randomized controlled trial. Interact Cardiovasc Thorac Surg.

[11] Wernovsky G, Wypij D, Jonas RA, Mayer JE, Hanley FL, Hickey PR (1995). Postoperative course and hemodynamic profile after the arterial switch operation in neonates and infants. A comparison of low-flow cardiopulmonary bypass and circulatory arrest. Circulation.

[12] Bronicki RA, Penny DJ, Anas NG, Fuhrman B (2016). Cardiopulmonary interactions. Pediatr Crit Care Med.

[13] Rochester DF, Bettini G (1976). Diaphragmatic blood flow and energy expenditure in the dog. Effects of inspiratory airflow resistance and hypercapnia. J Clin Invest.

[14] Green J, Walters HL, Delius RE, Sarnaik A, Mastropietro CW (2015). Prevalence and risk factors for upper airway obstruction after pediatric cardiac surgery. J Pediatr.

[15] Harrison AM, Cox AC, Davis S, Piedmonte M, Drummond-Webb JJ, Mee RBB (2002). Failed extubation after cardiac surgery in young children: prevalence, pathogenesis, and risk factors. Pediatr Crit Care Med.

[16] Ten Harkel ADJ, Van Der Vorst MMJ, Hazekamp MG, Ottenkamp J (2005). High mortality rate after extubation failure after pediatric cardiac surgery. Pediatr Cardiol.

[17] Walker SG, Stuth EA (2004). Single-ventricle physiology: perioperative implications. Semin Pediatr Surg.

[18] Sztrymf B, Messika J, Mayot T, Lenglet H, Dreyfuss D, Ricard JD (2012). Impact of high-flow nasal cannula oxygen therapy on intensive care unit patients with acute respiratory failure: a prospective observational study. J Crit Care.

[19] Mayfield S, Bogossian F, O’Malley L, Schibler A (2014). High-flow nasal cannula oxygen therapy for infants with bronchiolitis: pilot study. J Paediatr Child Health.

[20] Rubin S, Ghuman A, Deakers T, Khemani R, Ross P, Newth CJ (2014). Effort of breathing in children receiving high-flow nasal cannula. PediatrCrit Care Med.

[21] Pham TMT, O’Malley L, Mayfield S, Martin S, Schibler A (2015). The effect of high flow nasal cannula therapy on the work of breathing in infants with bronchiolitis. Pediatr Pulmonol.

[22] Kuwata S, Kurishima C, Kim J, Iwamoto Y, Saiki H, Ishido H (2015). Clinical evaluation of the hemodynamic effects of the high-flow nasal cannula therapy on the Fontan circulation. Clin Med Insights Cardiol.

[23] Habre W, Schütz N, Pellegrini M, Beghetti M, Sly PD, Hantos Z (2004). Preoperative pulmonary hemodynamics determines changes in airway and tissue mechanics following surgical repair of congenital heart diseases. Pediatr Pulmonol.

